# The role of serotonin inhibition within the treatment of carcinoid syndrome

**DOI:** 10.1530/EO-22-0077

**Published:** 2023-04-20

**Authors:** Joel George, John Ramage, Benjamin White, Rajaventhan Srirajaskanthan

**Affiliations:** 1 Hampshire Hospitals NHS Trust, Basingstoke, United Kingdom of Great Britain and Northern Ireland; 2 Kings Health Partners NET Centre of Excellence, London, United Kingdom of Great Britain and Northern Ireland

**Keywords:** carcinoids, neuroendocrine tumours, neuroendocrinology, somatostatin

## Abstract

Carcinoid syndrome is the most frequent hormonal complication associated with neuroendocrine neoplasms. It was first reported in 1954, and the classical symptoms are diarrhoea, flushing and abdominal pain. It is caused by the secretion of several vasoactive substances, the most prominent being serotonin, which play a pathophysiological role in the clinical symptoms which characterise carcinoid syndrome. Therefore, the focus of carcinoid syndrome treatment is to reduce serotonin production and hence improve the patient’s quality of life. There are a variety of management options for carcinoid syndrome including medical, surgical and loco-regional interventional radiological procedures. The most widely used are somatostatin analogues with three clinically approved drugs: lanreotide and octreotide (first-generation) and pasireotide (second-generation). Both everolimus and interferon used in combination with octreotide have shown significant reduction in urinary 5-hydroxyindoleacetic acid compared to octreotide alone. Telotristat ethyl has been increasingly utilised for patients with symptoms despite taking somatostatin analogues. It has also been shown to have a significant improvement in bowel movement frequency which was associated with a significant improvement in quality of life. Peptide receptor radionuclide therapy has proven symptomatic improvement in patients with uncontrolled symptoms. Chemotherapy is primarily reserved for patients with high proliferation tumours, with limited research on the efficacy in reducing symptoms. Surgical resection remains the optimal treatment due to being the only one that can achieve a cure. Liver-directed therapies are considered in patients where curative resection is not possible. There are therefore numerous different therapies. This paper describes the pathophysiology and therapy of carcinoid syndrome.

## Introduction

Neuroendocrine neoplasms (NENs) are neoplasms that arise from plenipotentiary neuroendocrine cells within the underlying organ. Under the most recent WHO classification, they are divided into well-differentiated neuroendocrine tumours (NETs) and poorly differentiated neuroendocrine carcinomas (NECs). There are four grades for NENs: NET G1 (Ki-67 index <3%), NET G2 (Ki-67 index 3–20%), NET G3 (Ki-67 index >20%) and NEC (Ki-67 index >20%). Ki-67 is a prognostic marker (Foltyn *et al.* 2012), expressed when cells are proliferating. They can also be categorised as foregut, midgut or hindgut depending on their embryonic origin. Foregut includes lung, stomach, duodenum and pancreas; midgut includes ileum, appendix and cecum and hindgut includes colon and rectum (Wang *et al.* 2019). NENs may secrete as many as 40 vasoactive substances, the most significant being serotonin (5-HT), but also tachykinins, histamine, prostaglandins and kallikrein (Ferrari *et al.* 2018). The presence of a clinical syndrome due to excessive hormone secretion, termed carcinoid syndrome (CS), denotes a functional NEN.

## Carcinoid syndrome

The commonest syndrome caused by functional NENs is CS. It has an estimated overall incidence of 1 in 100,000 persons (Halperin *et al.* 2017).

It was first described by Thorson *et al.* in 1954 as a new condition characterised by diarrhoea, pellagra-like skin lesions, peripheral vasomotor symptoms, valvular disease of the right side of the heart, an ‘unusual type of cyanosis’ and bronchoconstriction. These still make up the major clinical features of CS. The cardinal features to identify in a clinical setting are diarrhoea and flushing. Diarrhoea is reported in around 80% of patients. Patients with CS-related diarrhoea experience watery, loose stools multiple times a day (Kaltsas *et al.* 2004).

Flushing of the skin is seen in 50–85%, characterised by a rosy red appearance of the face, neck and the upper part of the chest, accompanied by a feeling of heat. A flush can last several minutes and can occur many times per day, leaving no permanent discolouration. It can appear spontaneously or be triggered by emotional stress, consumption of alcohol or tyramine-containing foods (e.g. chocolate, walnuts and bananas) (Kaltsas *et al.* 2004). Other common, clinically relevant symptoms of CS include abdominal pain (40%) and wheezing (15%).

CS is predominantly associated with NENs arising from the midgut with liver metastases but can be present in patients with bronchial or rarely in pancreatic NENs (Halperin *et al.* 2017) or pelvic disease bypassing the portosystemic circulation.

Symptomatic management of CS reduces 5-HT production in various ways with the purpose of reducing symptoms, improving quality of life (QoL) and delaying the development of sequelae.

## Diagnosis

The most easily accessible, validated diagnostic marker of CS is 24-h urine collection for 5-hydroxyindole acetic acid with approximately 70% sensitivity and 90% specificity (Fanciulli *et al.* 2020).

The accuracy of this marker is higher for midgut NENs due to greater production of 5-HT compared to hindgut and foregut. The level of 5-HIAA measured in the urine correlates with the tumour size; however, correction with clinical severity is weaker due to the inconsistent release of 5-HT from the tumours (Fanciulli *et al.* 2020).

However, there are restrictions with 24-h urine collections. First, it can be challenging for patients to collect all the urine over the 24-h period. In addition, consumption of tryptophan-rich foods (banana, chocolate, pineapple, etc.) or certain medications may give false-positive or false-negative results; hence, abstinence from these must be completed for 3 days prior to collection (Kaltsas *et al.* 2017). Plasma fasting 5-HIAA levels have been found to correlate with urinary 5-HIAA in patients with small intestine NENs while offering better reliability for follow-up (Tellez *et al.* 2013). Although it is a novel tool, it is likely that plasma 5-HIAA will be more prominently used for diagnosis in the future (Tellez *et al.* 2013). There is also spot urine collection for 5-HIAA which has several benefits including elimination of over- or under-collection of urine in addition to increased patient satisfaction. It has been shown there is a high correlation between spot urine 5-HIAA and 24-h urine 5-HIAA (Calanchini *et al.* 2019). It should be noted there are instances where a marked evaluation of urinary 5-HIAA is not seen in CS (Kaltsas *et al.* 2017).

## Serotonin

5-HT is an end product of the metabolism of the ingested essential amino acid tryptophan. It was characterised and isolated by Maurice Rapport and Irvine Page in 1948 (Rapport *et al.* 1948).

5-HT is a neurotransmitter which acts mainly in the central nervous system (CNS) and gastrointestinal tract. Approximately 95% of the total 5-HT is synthesised and released within the gastrointestinal tract by both enterochromaffin cells and enteric neurons. Meanwhile, the remaining 5% of the total 5-HT acts in the brain. 5-HT is primarily located in blood platelets, in serotonergic neurons, in intestinal myenteric plexus, and in enterochromaffin cells in the mucosa of the gastrointestinal tract where 5-HT is stored. Storage protects against degradation. 5-HT is converted to 5-hydroxyindole acetaldehyde (5-HIA) by the outer mitochondrial membrane enzyme monoamine oxidase (MAO). There are two molecular subtypes called MAO-A and MAO-B which are both widely prevalent in the brain and peripheral tissues.

5-HIA is readily metabolised by aldehyde dehydrogenase in the mitochondria to produce 5-HIAA, the major excreted urinary metabolite of 5-HT (Fanciulli *et al.* 2020). 5-HIA is also converted to 5-hydroxytryptophol by aldehyde reductase although this only accounts for around 1% of total 5-HT metabolism in normal conditions. Other minor metabolic pathways can be found in the liver, lung and brain.

Normally only 1% of dietary tryptophan is metabolised to 5-HT; however, in CS, the proportion is 70%. Blood 5-HT concentration is increased via 5-HT-secreting hepatic lesions which bypasses the liver to reach the systemic circulation.

Ovarian, testicular, retroperitoneal or bronchial primary NENs or metastases can also cause CS without the presence of liver metastases by accessing the systemic circulation directly.

Seven 5-HT receptor classes (5-HT1, 5-HT2, 5-HT3, 5-HT4, 5-HT5, 5-HT6 and 5-HT7) have been identified and are widely expressed throughout the body. 5-HT1, 5-HT2 and 5-HT5 all have subtypes. In addition, 5-HT3 is the only ligand-gated Na+/K+ ion channel receptor, and the remaining six are all G-protein-coupled receptors.

The effects of 5-HT are broad and diverse. Behavioural and neurophysiological processes modulated by 5-HT include but are not limited to perception, mood, reward, anger, appetite and memory (Berger *et al.* 2009). In vascular biology, 5-HT causes vasoconstriction or vasodilation in different vascular beds depending on the receptors expressed in the vessel wall (Berger *et al.* 2009).

5-HT regulates several aspects of cardiac function, from electrical conduction to valvular closure. In the respiratory system, it helps control breathing and respiratory drive through pulmonary vasculature and brainstem respiratory control centres. 5-HT modulates the activity of rhythm-generating respiratory neurons in the brainstem pre-Boetzinger complex via the 5-HT4 receptor (Berger *et al.* 2009). A high 5-HT is thought to be the main mediator of diarrhoea by acting directly on cell membrane receptors of enteric neurons. This increases peristalsis and impedes intestinal absorption. 5-HT may stimulate the synthesis of extracellular matrix (ECM), leading to fibrosis and resulting in bowel obstruction and carcinoid heart disease in the long term. There is a correlation between these complications and raised urine 5-HIAA (Ferrari *et al.* 2018).

The tumour microenvironment has been recognised as a critical determinant of tumour progression. Svedja *et al.* investigated the crosstalk between tumour cells and fibroblasts. It was found the addition of a specific 5-HT2B receptor antagonist in the tumour cell-containing compartment inhibited the proliferation of KRJ-I cells and significantly reduced 5-HT release, while also reducing the synthesis of transforming growth factor beta-1 (TGF-β1), connective tissue growth factor and fibroblast growth factor 2 (FGF2) (Svejda *et al.* 2010).

Fibroblasts are stimulated by 5-HT and by several growth factors including TGF-β. 5-HT and TGF-β induce connective tissue growth factors which increase collagen synthesis, fibroblast proliferation and differentiation into myofibroblasts. In addition, TGF-β stimulates the basic FGF2 and platelet-derived growth factor (PDGF). The final component of the tumour microenvironment is the ECM which provides biochemical and biomechanical cues for tissue homeostasis and structural support. TGF-β stimulates stromal cells to induce myofibroblastic differentiation and ECM remodelling and production. Myofibroblast cells secrete TGF-β creating a profibrotic feedback loop (Blažević *et al.* 2018).

## Management of serotonin and carcinoid syndrome

Management of CS can be achieved through a combination of medical, surgical and loco-regional interventional radiological procedures. Some of these therapies will have dual syndrome control and anti-tumour benefit.

### Somatostatin analogues

Somatostatin analogue (SSA) therapy has been a mainstay of antisecretory therapy in functioning NETs for several decades (Wolin 2012).

Somatostatin is a cyclic polypeptide which is processed into several peptide hormones, including SST-14 and SST-28. It is one of the main inhibitors of endocrine hormone secretion in humans; however, native somatostatin is not clinically useful due to the extremely short half-life (1–3 min). It is rapidly degraded by ubiquitously distributed peptidases in plasma and tissues (Benuck & Marks 1976). Synthetic SSAa with longer half-life were developed with three clinically approved drugs: lanreotide and octreotide (first-generation SSAs) and pasireotide (second-generation SSA).

Somatostatin inhibits the secretion of a variety of biological substances including growth hormone, gastric inhibitory peptide, gastrin and pancreatic polypeptide. It also inhibits the exocrine secretion of amylase, hydrochloric acid and pepsinogen and the absorption of glucose, fat and amino acids. Furthermore, somatostatin modulates gastrointestinal motility by delaying late-phase gastric emptying, weakening gallbladder contraction and prolonging small-intestine transit time.

Initially, it was used to inhibit the release of neuropeptides or bioactive amines. Beyond symptomatic management, recent research demonstrates that SSAs exert antiproliferative effects and inhibit tumour growth via the somatostatin receptor 2 (SSTR2). Direct antiproliferative effects occur through the activation of Src homology 2 domain phosphatase-1 and Src homology 2 domain phosphatase-2, which through a phosphorylation pathway, dephosphorylates phosphatidylinositol 3-kinase and extracellular signal-regulated protein kinases 1 and 2, leading to impaired cell proliferation. There are also indirect antiproliferative effects; through the inhibition of circulating growth factors (e.g. insulin-like growth factor 1 and vascular endothelial growth factor) and through the inhibition of tumour angiogenesis by altering the release of nitric oxide (Gomes-Porras *et al.* 2020).

Data suggested significantly better outcomes for patients with NETs between 1988 and 2004, compared to patients treated between 1973 and 1987. One possible cause is the introduction of octreotide as a treatment for patients with NETs (Yao *et al.* 2008). A study by Kvols *et al.* on 25 patients with CS with octreotide (three times a day subcutaneously) found that nearly 90% of patients reported a significant improvement in symptoms. In addition, 72% of patients had a 50% reduction of 24-h urine 5-HIAA (Kvols *et al.* 1986). Meta-analysis showed that octreotide reduced flushing and diarrhoea in 72 and 65% of patients, although lanreotide showed similar effects (Hofland *et al.* 2019). The PROMID and CLARINET studies were conducted in patients with midgut and gastroenteropancreatic NETs.

The PROMID study was a randomised, placebo-controlled, double-blind study in patients with well-differentiated metastatic midgut NETs, investigating the effect of long-acting octreotide 30 mg injected intramuscularly once a month, compared to the placebo. It concluded that long-acting octreotide significantly lengthens the time to tumour progression; however, overall survival (OS) analysis was not confirmatory. The median time to tumour progression in the long-acting octreotide group was 14.3 months compared to 6 months for the placebo group. After 6 months of treatment, 67% of patients showed stable disease compared to 37% of patients treated with placebo (Rinke *et al.* 2009).

The CLARINET study investigated the effect of long-vacting lanreotide compared to placebo in patients with advanced, G1 or G2 differentiated, non-functioning, somatostatin receptor-positive NETs and documented disease progression status. The administration of lanreotide was associated with significantly prolonged progression-free survival (PFS): at 24 months, 65% in the lanreotide group vs 33% for placebo (Caplin *et al.* 2014). Half of the patients in the lanreotide group had adverse effects related to the drug, with the most common adverse effect diarrhoea (26%).

5-HIAA levels were lower in 45–46% of CS patients treated with either lanreotide or octreotide compared to only 29% of patients treated with pasireotide (Hofland *et al.* 2019).

Conventional SSAs bind to the SSTR2. However, pasireotide is a novel multireceptor-targeted somatostatin that also binds to somatostatin receptor 1, 3 and 5 in addition to SSTR2. Preclinical data indicate a more potent antiproliferative effect compared to octreotide, giving rise to optimism that pasireotide could represent a more effective antiproliferative tool in the treatment of patients with NETs.

A phase II study assessed the clinical activity of pasireotide in patients with metastatic grade 1 or 2 NEN. A total of 29 patients were treated with 60 mg pasireotide long-acting release (LAR) every 4 weeks. The median PFS was 11 months. However, the median OS was not reached; the 30-month OS was 70%. The treatment was associated with a 79% rate of hyperglycaemia, questioning suitability as a first-line treatment (Cives *et al.* 2015).

A phase III study compared pasireotide LAR with octreotide LAR in managing carcinoid symptoms refractory to first-generation SSAs. Patients with carcinoid tumours of the digestive tract were randomly assigned to either receive 60 mg pasireotide LAR or 40 mg octreotide LAR every 28 days. Pasireotide LAR prolonged median PFS by 5 months. The most frequent drug-related adverse effects were hyperglycaemia, fatigue and nausea, which although higher in the pasireotide group were not significant (Wolin *et al.* 2015).

### Telotristat ethyl

**Figure 1:**
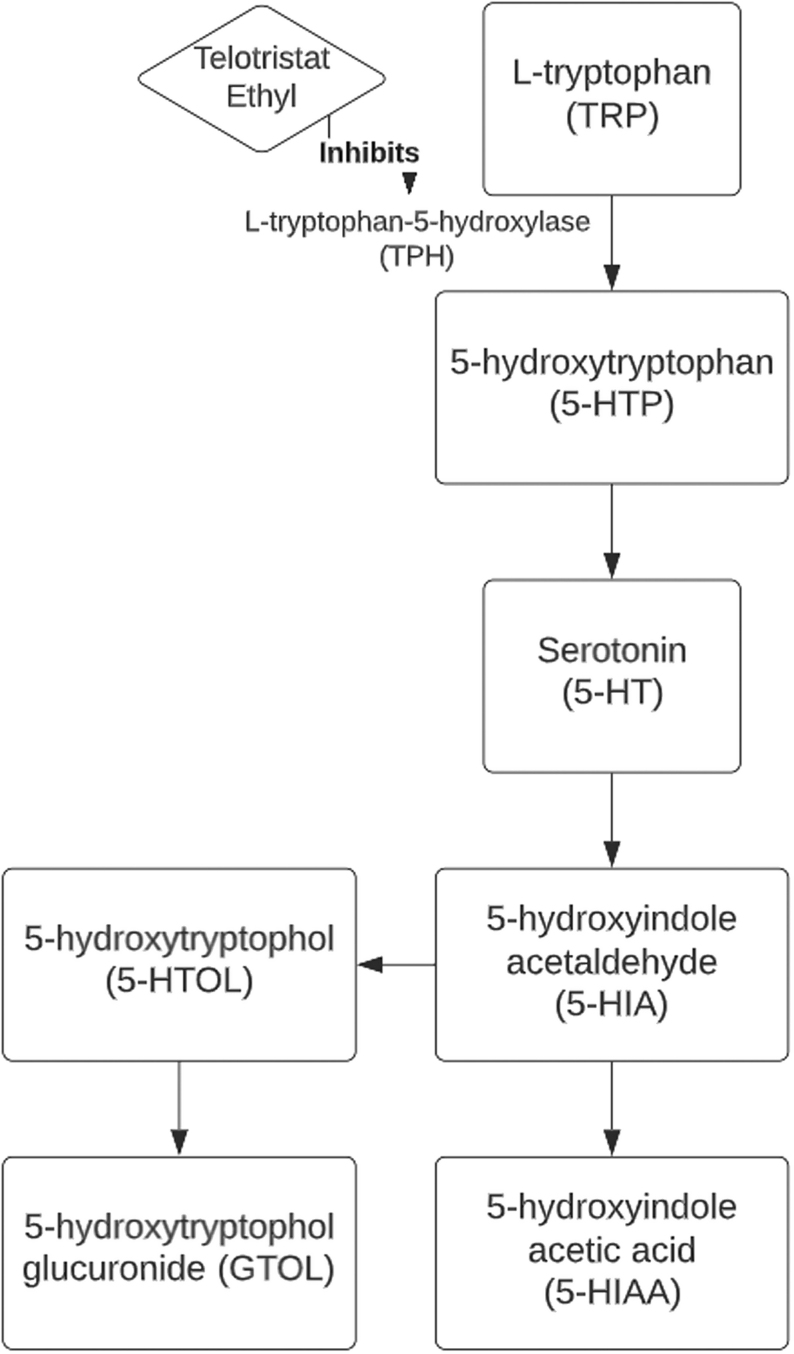
Role of telotristat ethyl in 5-HT inhibition.

Another therapeutic option for controlling symptoms in CS is the inhibition of the peripheral synthesis of 5-HT (Fig. 1). The molecular target is TPH, the rate-limiting enzyme in the pathway. There are two isoforms of TPH (TPH1 and TPH2) each with unique tissue specificity. TPH1 is primarily located in the enterochromaffin cells and is part of the pathogenesis of gastrointestinal symptoms of CS. Meanwhile, TPH2 is exclusively expressed in the CNS in both the myenteric plexus and the brain. TPH 1 and 2 play an important role in gastrointestinal motility and mood regulation. As a result, non-specific inhibition of TPH can have adverse CNS effects.

This hypothesis was tested by Engelman *et al.* using parachlorophenylalanine in 1967. In the study, urinary 5-HIAA levels fell and symptoms improved. However, the drug caused severe CNS-related adverse effects including depression (Engelman *et al.* 1967).

Telotristat ethyl (TE) is a novel, oral, tryptophan hydroxylase inhibitor. It has a high-molecular-weight and acidic moieties, inhibiting the drug from crossing the blood–brain barrier, thus creating a ‘physiological’ selectivity for TPH1; TE produces a significant reduction in 5-HT concentration in the intestine without impacting the concentration of 5-HT in the CNS (Saavedra *et al.* 2019).

Telotristat etiprate is the hippurate salt of TE. TE is approved for the treatment of refractory CS diarrhoea; it was stated that patients with a minimum of four daily bowel movements benefit from treatment.

Peak plasma concentration of TE was achieved between 30 min to 2 h. The drug is metabolised by carboxylesterases extensively into the active metabolite, telotristat. The peak concentration of telotristat was within 1–3 h. Afterwards, the plasma concentrations declined in a biphasic manner.

TELECAST phase 3 trial assessed the safety and efficacy of TE in patients with CS during a 12-week double-blind treatment period followed by a 36-week open-label extension (OLE). A total of 76 patients were randomly assigned to receive either placebo, 250 mg TE or 500 mg TE three times per day. At week 12, a significant reduction in urinary 5-HIAA was observed for both 250 mg TE and 500 mg TE compared to the placebo. This reduction continued during the OLE period. Treatment-related adverse events were generally mild to moderate in severity and were most commonly gastrointestinal in nature. The overall incidence of any TRAE was similar in both placebo and TE groups. There was no dose-dependent or drug-dependent relationship between TE and gastrointestinal symptoms. There was no increase in depression-related adverse effects in both the double-blind treatment and the OLE.

The study showed 75% of patients on TE experienced a significant improvement in bowel movement frequency by 12 weeks. In addition, improvements in stool form were observed (Dillon *et al.* 2021). It was recommended to take TE with meals as a high-fat meal improved systemic exposure with a peak plasma concentration 112% greater compared with fasted (Dillon *et al.* 2021). Improvement in bowel movement frequency was associated with significant improvements in QoL. Patients reported improvements in social and physical functioning as well as emotional well-being (Cella *et al.* 2018).

A weight increase has been observed in patients taking TE. Up to 32.5% of patients experienced significant, dose-dependent weight gain. Weight gain coincided with improved biochemical and metabolic parameters and fewer serious adverse events compared to those whose weight remained the same or reduced (Weickert *et al.* 2018). A 57% decrease in urinary 5-HIAA was observed in patients who experienced weight gain, with a mean change in urinary 5-HIAA of −90 mg/24 h at week 12.

### Surgery

Surgical resection remains the optimal treatment for NEN since this is the only treatment that can achieve a cure. Surgical intervention is to remove the entire tumour where possible and debulk any metastases. Only 10–20% of patients with hepatic NETs with liver metastases are candidates for surgery (Jones *et al.* 2012).

Although survival was prolonged in the subset of patients who were able to undergo potentially curative resections, symptom improvement has been observed in patients undergoing curative and palliative resections (Palade *et al.* 2019).

Norton conducted a study on 16 post-surgical patients with NETs with liver metastases, 8 of whom had CS. It recommended early, extensive surgical resection in patients with NETs in the liver. However, other treatments would be required to decrease the likelihood of tumour recurrence. In addition, the small sample size and short follow-up limits the impact of the findings (Norton *et al.* 2003).

Another study showed the likelihood for recurrence for NETs reduces for completely resected liver metastases; however, recurrence remained high at 76% with a median time to recurrence of 30 months, compared to the 91% and 16 months for the incompletely resected group (Gomez *et al.* 2007). Although complete resection was an important factor for influencing recurrence, the high recurrence rates favours performing more incomplete resections to preserve more liver parenchyma.

Another alternative approach with curative intent is liver transplantation. Transplantation for NET is an area with a growing interest and body of research. It is offered to those whose liver disease is not amenable to resection. Research reported a 5-year OS of 80% for transplantation for NET hepatic metastases; however, recurrence was diagnosed in 12 of the 19 patients between 2 weeks and 48 months after liver transplantation (Rosenau *et al.* 2002).

Resection of mesenteric metastases may alleviate symptoms and prolong survival. In the research by Soreide *et al.*, the median survival of patients who underwent resection of midgut primary carcinoids and mesenteric metastases was 139 months compared to 69 months when no debulking was performed (Sutton *et al.* 2003).

### Peptide receptor radionuclide therapy

Peptide receptor radionuclide therapy (PRRT) is an effective form of radioligand therapy. The aim of this therapy is to target receptors overexpressed on tumours (e.g. SSTR2), leading to the irradiation of tumour cells and the surrounding blood vessels and inhibiting angiogenetic response (Basu *et al*. 2020). Patients receive an IV administration of a radioactive substance which is bound covalently to a hormone analogue of high affinity and specificity to SSTRs on tumour cells.

The most common treatment is four PRRT cycles spaced approximately 8 weeks apart. Before the radioactive drug is injected, pre-medications of anti-nausea and an amino acid solution are administered to help with the potential nausea and vomiting during treatment and to protect the patient’s kidney from absorbing radiation. After the radioactive drip, the amino acid infusion is continued.

A multinational phase III randomised, double-blind, prospective clinical trial evaluated the efficacy and safety of PRRT (^177^Lu-DOTA-TATE) in patients with advanced, progressive, somatostatin receptor–positive midgut NENs. A total of 229 patients were randomly assigned to receive ^177^Lu-DOTA-TATE or high-dose LAR octreotide therapy. Results from this study showed that ^177^Lu-DOTA-TATE (with octreotide-LAR 30 mg) significantly prolonged PFS compared to octreotide-LAR alone, in patients with well-differentiated metastatic midgut NENs. PRRT was associated with limited acute toxic effects during the observed time period (Camus *et al.* 2021). This paved the way for the approval of Lutathera® (^177^Lu-DOTA-TATE) by the EMA in 2017 and the FDA in 2018 (Hennrich & Kopka 2019).

^177^Lu-DOTATATE was associated with the highest OS and the lowest rate of adverse events when directly compared to ^90^Y-DOTATOC. However, the combination of ^177^Lu-DOTATATE and ^90^Y-DOTATOC proved to be the most effective in reducing tumour size and in controlling tumour growth. An explanation could be a synergistic anti-tumour effect due to the different physical properties of both (Camus *et al.* 2021).

^177^Lu-DOTATATE has proven symptomatic improvement in patients with uncontrolled symptoms of CS. A study showed that after PRRT, both the bowel movement frequency and daily flushing reduced. In 47% of patients with a baseline bowel movement frequency of four or more, a decrease of >30% was observed. In addition, 67% of patients who experienced two or more episodes of daily flushing had a reduction of >50% in flushing. These improvements in symptoms were associated with a reduction of urinary 5-HIAA. In 18 patients with follow-up of more than 6 months, the median 24-h urinary 5-HIAA excretion decreased by 245 µmol/24 h (775 µmol/24 h to 530 µmol/24 h (Zandee *et al.* 2021)).

PRRT has a number of adverse effects, such as haematological and less commonly renal, hepatic toxicity. A serious long-term adverse effect is myelodysplastic syndrome which was reported at 0.18–0.79% in varying studies (Camus *et al.* 2021).

Scintigraphy scans are performed to confirm that the tumour co-localises with binding to the specific ligand used during PRRT. Although infrequent, carcinoid crises can occur during or even a few days after the administration of PRRT.

The first case of a carcinoid crisis induced by PRRT was believed to be reported by Davi *et al.* (Davì *et al.* 2006). In this case, the patient experienced severe carcinoid symptoms (profuse diarrhoea, persistent flushing and loss of appetite) and hence was hospitalised. An i.v. infusion of octreotide (50 μg/h) along with H1 and H2 receptor antagonists and corticosteroids eventually controlled the symptoms after a few days (Tapia Rico *et al.* 2018).

### Everolimus

Everolimus is a derivative of sirolimus (rapamycin) bearing a 2-hydroxyethyl chain at position 40. Sirolimus is a macrolide antibiotic; hence like sirolimus, everolimus has potent immunosuppressive and antiproliferative effects but with greater solubility and stability (Böhler *et al.* 1998).

A series of phase II/III clinical studies were conducted to assess the efficacy and safety of everolimus in patients with advanced NENs.

RADIANT 2 was a randomised, double-blind, placebo-controlled, phase III trial. A total of 429 patients who presented with low- or intermediate-grade metastatic or unresectable NEN with CS were randomly assigned to either everolimus (10 mg/day) or placebo with both groups receiving octreotide LAR (30 mg every 28 days). Patients treated with everolimus and octreotide LAR showed an improvement of 5.1 months in median PFS compared with the placebo and octreotide LAR (16.4 months vs 11.3 months); however, it marginally missed the threshold for statistical significance. Everolimus and octreotide LAR demonstrated a greater reduction in 24-h urinary 5 HIAA levels compared to the placebo and octreotide LAR (Pavel *et al.* 2011).

A sub-analysis of the RADIANT-2 study reporting the final (OS) from the study concluded that the combination of everolimus and octreotide LAR did not extend OS compared with octreotide LAR alone. Multiple factors including unbalanced patient characteristics and uneven SSA exposure likely influenced the survival outcome (Pavel *et al.* 2017). Everolimus is only licensed for the treatment of non-functional NETs (Yao *et al.* 2011).

### Lenvatinib

Lenvatinib is a receptor tyrosine kinase inhibitor that inhibits the kinase activities of vascular endothelial growth factor receptor 1–3 (VEGFR1–3), fibroblast growth factor receptor 1–4 (FGFR1–4) and PDGF receptor alpha and beta. In particular, it has a high potency against FGFR1 – a key driver of resistance to antiangiogenic drugs.

Treatment-related resistance is common in patients treated with VEGF inhibitors; however, the available data suggest that lenvatinib may increase the efficacy of others and could even revert primary and acquired resistance.

TALENT was a multicenter, single-arm, open-label phase II trial. A total of 111 patients were enrolled who had histologically confirmed advanced grade 1–2 pancreatic NET or gastrointestinal NET with documented tumour progression after treatment with a molecular-targeted agent or SSAs. Treatment consisted of oral lenvatinib 24 mg once daily until treatment intolerance or disease progression. Patients treated with lenvatinib had a median PFS of 15.7 months, with both groups producing similar outcomes and with results favourable compared to the RADIANT trials with everolimus. However, the lack of randomisation comparing lenvatinib with an alternative treatment is the main limitation of the trial (Capdevila *et al.* 2021).

### Interferon

Interferon (IFN) was discovered in 1957 and was later identified as a group of related protein cytokines secreted by cells in response to stimulation by a virus or other foreign substance (Murren & Buzaid 1989).

Several studies have proven that IFN-α reduces symptoms as well as tumour markers and tumour growth in patients suffering from CS.

A study by Janson *et al.* on the long-term management of CS comparing treatment with octreotide alone and in combination with IFN-α concluded that a combination of octreotide and IFN-α is most beneficial for patients since it demonstrated additive clinical effects without any severe toxicity. Of the 19 patients who had the combination of IFN-α and octreotide, 72% showed significant reduction in U-5HIAA for a median of 10 months. The commonest side effects associated with interferon were flu-like symptoms (74%), tiredness and muscle pain (53%), anorexia and/or weight loss (Janson & Öberg 1993).

The majority of IFN side effects are dose-dependent, more noticeable in the elderly, and reversible when treatment is stopped.

Side effects of IFN are subacute or chronic. The most common adverse effect is an acute influenza-like syndrome visible in >95% of patients. Symptoms usually decrease in intensity and disappear within 7–10 days of continued therapy in patients who receive IFN at least three times a week; however, it can reoccur if therapy is interrupted. Other side effects include cardiovascular, gastrointestinal, renal, neurological, haematological and dermatological. In practice, IFN is rarely used now in view of the side effects.

### Chemotherapy

Chemotherapy uses drugs which are injected intravenously and intramuscularly or taken orally to kill cancer cells, travelling through the blood to reach cancer cells throughout the body.

Chemotherapy should be primarily reserved for patients with high proliferation tumours, except possibly for pancreatic primary sites Ki-67 of >10%, and widespread disease might support systemic chemotherapy as a first-line treatment (Öberg 2001). There are limited data regarding the efficacy of chemotherapy in reducing CS symptoms.

One chemotherapy agent is temozolomide (TMZ), an alkylating agent, which is a less toxic and oral derivative of intravenous dacarbazine. TMZ has a bioavailability of approximately 100% after oral administration; however, it is metabolised quickly and eliminated. It is a prodrug that works by undergoing spontaneous hydrolysis in the presence of slightly alkaline pH values to its active compound which further degrades to reactive methyldiazonium ions. This can cause methylation, resulting in base pair mismatch which damages the DNA. When mismatch repair enzymes attempt to remove the adduct, they produce breaks in the DNA, triggering cell death via apoptosis. TMZ has shown antitumour activity both in combination with other drugs and as a single agent (Koumarianou *et al.* 2015).

A recent study on 43 patients with advanced pancreatic NET tested the combination of TMZ (taken on days 1–7 and days 15–21) and everolimus daily in a 28-day cycle for 6 months. The PFS was 15.4 months, but the efficacy of the combination compared to TMZ alone has not been formally evaluated (Chan *et al.* 2013).

### Liver-directed therapies

Surgery of liver metastases can be considered for some patients as this offers the only chance of cure. However, for many patients, curative resection is not possible, and so liver-directed embolisation therapies may be considered.

The 5-year survival rates for patients treated with hepatic arterial embolisation (otherwise known as transarterial hepatic embolisation, TAE) was 50%, compared to 76% for patients who underwent surgery (Chamberlain *et al.* 2000). However, surgery cannot be proposed for all patients with liver metastases secondary to NETs. TAE can be recommended in patients who are considered nonsurgical candidates and have vascular metastases. The rationale is that most liver metastases are hyper-vascular and derive their blood supply from the hepatic artery. The aim of the procedure is to induce ischaemia of tumour cells, hence reducing hormone output and causing necrosis using various particulates or microspheres. In 64 patients with midgut CS and disseminated liver metastases, it was effective in reducing symptoms with a 55% reduction in 5-HIAA levels 71 ± 11 months of follow-up.

Transarterial chemoembolisation (TACE) was developed based on the principle that ischaemia of tumour cells increases sensitivity to chemotherapeutic substances. Studies have compared TAE and TACE in patients with liver metastases. Gupta *et al.* found that TACE did not improve OS or PFS in patients with small intestinal tumours compared to TAE (Gupta 2013).

A study by Drougas *et al.* conducted a study where patients who demonstrated symptom progression despite treatment with SSAs were given intra-arterial chemotherapy and hepatic artery chemoembolisation. 5-HIAA reduced by 60 ± 6% s.e. at 3 months and symptoms of diarrhoea, flushing and abdominal pain were improved in the majority of patients, confirming that hepatic artery embolisation improves symptoms of CS and improves short-term QoL in patients with advanced hepatic carcinoid disease (Gupta 2013).

Side effects of embolisation include abdominal pain, nausea, fever and an increase in liver enzymes (such as serum transaminases which increase significantly, peaking 2–3 days after embolisation). The majority of symptoms resolve within 1 week of treatment (Strosberg *et al.* 2011).

Radioembolisation with yttrium-90 microspheres involves the infusion of embolic microparticles of glass or resin impregnated with the isotope yttrium-90 through a catheter directly into the hepatic arteries. Yttrium-90 is a beta emitter and has a physical half-life of 64.1 h. Patients with mild to moderate liver dysfunction or portal vein thrombosis who are ineligible for chemoembolisation or bland embolisation may be able to tolerate radioembolisation (SIRT). In addition, the toxicities associated with radioembolisation appear lower compared to other embolisation techniques since it does not induce ischemic hepatitis. A rare but serious complication is radiation enteritis, which occurs when particles accidentally infuse into arteries supplying the gastrointestinal tract (Strosberg *et al.* 2011). In a study of 148 patients treated with resin microsphere, the median survival was 70 months (Gupta 2013). However, SIRT is not licensed in NETs.

Thermal ablative ablation is based on the cytotoxic effects of non-physiologic temperature that are locally administered by probes placed within the liver. Various devices have been developed to ablate tumours using heat. One of the most popular methods is microwave ablation (MWA) which can be facilitated via imagine-guided percutaneous, laparoscopic or open surgical approaches. It can be a single procedure or combined with other modalities. MWA uses electromagnetic microwaves (with frequencies ≥900 MHz) to agitate water molecules in the surrounding tumour tissue causing cell death through coagulation necrosis. One study reported that in 11 patients with liver metastases who underwent MWA, there was no local liver recurrence (Martin *et al.* 2010).

## Carcinoid crisis

A serious complication of CS is carcinoid crisis, an exacerbation of CS which can potentially be life-threatening. Symptoms include severe flushing, bronchospasm, profound hypotension and arrhythmias or hypertension, CNS dysfunction (stupor and confusion) and diarrhoea.

It was first reported by Kahil *et al.* in 1964 when a woman started to manifest episodes of flushing, cramps and pruritus a few months after she underwent surgical resection of a NEN of the ileum (Kahil *et al.* 1964). She was treated with cyproheptadine (peripheral 5-HT antagonist) and obtained good control of her symptoms. However, 13 days after discontinuation of therapies, she manifested further symptoms, including sudden onset oppressive chest pain, abdominal cramps, frequent diarrhoea, facial flushing, progressive extreme hypotension, pruritis and peripheral vascular collapse. A single i.v. injection of cyproheptadine was administered an hour after the onset of symptom, followed by a dramatic cessation of the chest and abdominal pain. It was suggested the sudden release of active substances by neuroendocrine cells provoked by stress causes this complication.

Carcinoid crisis appears spontaneously but can be precipitated by surgery, PRRT, chemotherapy, radiological procedures and stress. Octreotide intravenously is advised to prevent and treat carcinoid crisis (Kaltsas *et al.* 2017).

## Impact of CS on daily life and healthcare cost

Patients with NETs have high rates of cognitive impairment, depression, impairment in sleep, fatigue, anxiety and a decrease in overall physical function (Fröjd *et al.* 2007). An increased CS burden (measured by a higher frequency of bowel movements and flushing, greater number of symptoms and impaired activity levels) was associated with lower QoL. Findings demonstrated even a low frequency of an event like flushing, which might be dismissed by the clinician can have a profound effect on the QoL (Pearman *et al.* 2016).

Diarrhoea has a particularly prominent adverse effect on physical, emotional and social well-being (Fröjd *et al.* 2007).

Broder *et al.*, in a retrospective cohort study, demonstrated that healthcare costs and resources required in newly diagnosed CS patients with diarrhoea are consistently and significantly higher than in those without diarrhoea (Broder *et al.* 2016).

Another retrospective cohort study by Dasari *et al.* supported previous studies since it concluded that the economic burden of CS diarrhoea is greater than that of CS alone among insured working-age adults in the United States. The total adjusted annual healthcare cost per patient was 50% higher among those with CS diarrhoea, mainly driven by outpatient services, prescriptions and emergency department visits (Dasari *et al.* 2019).

CS negatively impacts the patient and the healthcare system. Timely identification and management of CS and CS-related diarrhoea may reduce the economic burden.

## Conclusion and future perspectives

CS is the most frequent hormonal complication associated with NENs. It is caused by the secretion of several vasoactive substances, the most prominent being 5-HT, which play a pathophysiological role in the clinical symptoms which characterise CS. The major acute life-threatening complication of CS is carcinoid crisis. CS has been shown to have a significant impact on patients’ QoL. The aim of CS treatment is to reduce 5-HT production, control symptoms and improve QoL.

There are a variety of treatment options for CS, the most widely used being SSAs, with TE increasingly utilised more recently. First-line management is usually SSAs with other management options (including debulking surgery, TAE/TACE, PRRT and IFN-α) generally used for refractory CS, defined by recurring or persisting CS symptoms and increasing or persistently high u5-HIAA levels despite use of maximum doses of SSA.

Therapeutic options should be discussed within an multidisciplinary team (MDT), with the choice of treatment depending on if it is a radiologically stable or progressive NET. Due to the development of symptomatic progression while on SSA, symptom relief should be monitored initially monthly and then once symptom relief is achieved, monitoring should be done every 4–6 months.

Choice of 5HT inhibition should be personalised to the patient and plays a key role in the treatment of CS, patient outcomes and the resultant cost to the wider health system.

Future perspectives focussed on whether earlier aggressive management of CS could reduce the risk of development of the complications of CS would be important.

## Declaration of interest

The authors have no conflicting interests to declare.

## Funding

This work did not receive any specific grant from any funding agency in the public, commercial or not-for-profit sector.

## Author contribution statement

JG writing and literature search, JKR writing, RS and BW writing and review of manuscript.

## References

[bib1] Basu S Parghane RV Kamaldeep & Chakrabarty S 2020 Peptide receptor radionuclide therapy of neuroendocrine tumors. Seminars in Nuclear Medicine 50 447–464. (10.1053/j.semnuclmed.2020.05.004) 32768008

[bib2] Benuck M & Marks N 1976 Differences in the degradation of hypothalamic releasing factors by rat and human serum. Life Sciences 19 1271–1276. (10.1016/0024-3205(7690263-0) 825697

[bib3] Berger M Gray JA & Roth BL 2009 The expanded biology of serotonin. Annual Review of Medicine 60 355–366. (10.1146/ANNUREV.MED.60.042307.110802) PMC5864293 19630576

[bib4] Blažević A Hofland J Hofland LJ Feelders RA & De Herder WW 2018 Small intestinal neuroendocrine tumours and fibrosis: an entangled conundrum. Endocrine-Related Cancer 25 R115–R130. (10.1530/ERC-17-0380) 29233841

[bib5] Böhler T Waiser J Budde K Lichter S Jauho A Fritsche L Korn A & Neumayer HH 1998 The in vivo effect of rapamycin derivative SDZ RAD on lymphocyte proliferation. Transplantation Proceedings 30 2195–2197. (10.1016/S0041-1345(9800588-0) 9723438

[bib6] Broder MS Chang E Romanus D Cherepanov D & Neary MP 2016 Healthcare and economic impact of diarrhea in patients with carcinoid syndrome. World Journal of Gastroenterology 22 2118–2125. (10.3748/wjg.v22.i6.2118) 26877616 PMC4726684

[bib7] Calanchini M Tadman M Krogh J Fabbri A Grossman A & Shine B 2019 Measurement of urinary 5-HIAA: correlation between spot versus 24-h urine collection. Endocrine Connections 8 1082–1088. (10.1530/EC-19-0269) 31265996 PMC6652243

[bib8] Camus B Cottereau AS Palmieri LJ Dermine S Tenenbaum F Brezault C & Coriat R 2021 Indications of peptide receptor radionuclide therapy (Prrt) in gastroenteropancreatic and pulmonary neuroendocrine tumors: an updated review. Journal of Clinical Medicine 10 1–18. (10.3390/jcm10061267) PMC8003169 33803817

[bib9] Capdevila J Fazio N Lopez C Teulé A Valle JW Tafuto S Custodio A Reed N Raderer M Grande E, 2021 Lenvatinib in patients with advanced Grade 1/2 pancreatic and gastrointestinal neuroendocrine tumors: results of the Phase II Talent trial (GETNE1509). Journal of Clinical Oncology 39 2304–2312. (10.1200/JCO.20.03368) 33945297

[bib10] Caplin ME Pavel M Ćwikła JB Phan AT Raderer M Sedláčková E Cadiot G Wolin EM Capdevila J Wall L, 2014 Lanreotide in metastatic enteropancreatic neuroendocrine tumors. New England Journal of Medicine 371 224–233. (10.1056/NEJMoa1316158) 25014687

[bib11] Cella D Beaumont JL Hudgens S Marteau F Feuilly M Houchard A Lapuerta P Ramage J Pavel M Hörsch D, 2018 Relationship between symptoms and health-related quality-of-life benefits in patients with carcinoid syndrome: post hoc analyses from TELESTAR. Clinical Therapeutics 40 2006–2020.e2. (10.1016/j.clinthera.2018.10.008) 30477789

[bib12] Chamberlain RS Canes D Brown KT Saltz L Jarnagin W Fong Y & Blumgart LH 2000 Hepatic neuroendocrine metastases: does intervention alter outcomes? Journal of the American College of Surgeons 190 432–445. (10.1016/S1072-7515(0000222-2) 10757381

[bib13] Chan JA Blaszkowsky L Stuart K Zhu AX Allen J Wadlow R Ryan DP Meyerhardt J Gonzalez M Regan E, 2013 A prospective, phase 1/2 study of everolimus and temozolomide in patients with advanced pancreatic neuroendocrine tumor. Cancer 119 3212–3218. (10.1002/CNCR.28142) 23733618 PMC4308727

[bib14] Cives M Kunz PL Morse B Coppola D Schell MJ Campos T Nguyen PT Nandoskar P Khandelwal V & Strosberg JR 2015 Phase II clinical trial of pasireotide long-acting repeatable in patients with metastatic neuroendocrine tumors. Endocrine-Related Cancer 22 1–9. (10.1530/ERC-14-0360) 25376618 PMC4643672

[bib15] Dasari A Joish VN Perez-Olle R Dharba S Balaji K & Halperin DM 2019 Direct costs of carcinoid syndrome diarrhea among adults in the United States. World Journal of Gastroenterology 25 6857–6865. (10.3748/wjg.v25.i47.6857) 31885426 PMC6931008

[bib16] Davì MV Bodei L Francia G Bartolomei M Oliani C Scilanga L Reghellin D Falconi M Paganelli G Lo Cascio V, 2006 Carcinoid crisis induced by receptor radionuclide therapy with 90Y-DOTATOC in a case of liver metastases from bronchial neuroendocrine tumor (atypical carcinoid). Journal of Endocrinological Investigation 29 563–567. (10.1007/BF03344149) 16840837

[bib17] Dillon JS Kulke MH Hörsch D Anthony LB Warner RRP Bergsland E Welin S O’Dorisio TM Kunz PL McKee C, 2021 Time to sustained improvement in bowel movement frequency with telotristat ethyl: analyses of Phase III studies in carcinoid syndrome. Journal of Gastrointestinal Cancer 52 212–221. (10.1007/s12029-020-00375-2) 32146619 PMC7714089

[bib18] Engelman K Lovenberg W & Sjoerdsma A 1967 Inhibition of serotonin synthesis by para-chlorophenylalanine in patients with the carcinoid syndrome. New England Journal of Medicine 277 1103–1108. (10.1056/NEJM196711232772101) 6054996

[bib19] Fanciulli G Ruggeri RM Grossrubatscher E Calzo Lo F Wood TD Faggiano A Isidori A & Colao A 2020 Serotonin pathway in carcinoid syndrome: clinical, diagnostic, prognostic and therapeutic implications. Reviews in Endocrine and Metabolic Disorders 21 599–612. (10.1007/S11154-020-09547-8/TABLES/6) 32152781

[bib20] Ferrari AC Glasberg JP & Riechelmann RP 2018 Carcinoid syndrome: update on the pathophysiology and treatment. Clinics 73(Supplement 1) e490s. (10.6061/clinics/2018/e490s) 30133565 PMC6096975

[bib21] Foltyn W Zajeçki W Marek B Kajdaniuk D Sieminśka L Zemczak A & Kos-Kudał B 2012 The value of the Ki-67 proliferation marker as a prognostic factor in gastroenteropancreatic neuroendocrine tumours. Endokrynologia Polska 63 362–366. 23115069

[bib22] Fröjd C Larsson G Lampic C & Von Essen L 2007 Health related quality of life and psychosocial function among patients with carcinoid tumours. A longitudinal, prospective, and comparative study. Health and Quality of Life Outcomes 5 18. (10.1186/1477-7525-5-18) 17428340 PMC1852299

[bib23] Gomes-Porras M Cárdenas-Salas J & Álvarez-Escolá C 2020 Somatostatin analogs in clinical practice: a review. International Journal of Molecular Sciences 21 1682. (10.3390/IJMS21051682) 32121432 PMC7084228

[bib24] Gomez D Malik HZ Al-Mukthar A Menon KV Toogood GJ Lodge JPA & Prasad KR 2007 Hepatic resection for metastatic gastrointestinal and pancreatic neuroendocrine tumours: outcome and prognostic predictors. HPB 9 345–351. (10.1080/13651820701504199) 18345317 PMC2225511

[bib25] Gupta S 2013 Intra-arterial liver-directed therapies for neuroendocrine hepatic metastases. Seminars in Interventional Radiology 30 28–38. (10.1055/s-0033-1333651) 24436515 PMC3700796

[bib26] Halperin DM Shen C Dasari A Xu Y Chu Y Zhou S Shih YT & Yao JC 2017 Frequency of carcinoid syndrome at neuroendocrine tumour diagnosis: a population-based study. Lancet. Oncology 18 525–534. (10.1016/S1470-2045(1730110-9) 28238592 PMC6066284

[bib27] Hennrich U & Kopka K 2019 Lutathera®: the first FDA-and EMA-approved radiopharmaceutical for peptide receptor radionuclide therapy. Pharmaceuticals 12 114. (10.3390/ph12030114) 31362406 PMC6789871

[bib28] Hofland J Herrera-Martínez AD Zandee WT & De Herder WW 2019 Management of carcinoid syndrome: a systematic review and meta-analysis. Endocrine-Related Cancer 26 R145–R156. (10.1530/ERC-18-0495) 30608900

[bib29] Janson ET & Öberg K 1993 Long-term management of the carcinoid syndrome treatment with octreotide alone and in combination with alpha-interferon. Acta Oncologica 32 225–229. (10.3109/02841869309083916) 7686765

[bib30] Jones NB Shah MH & Bloomston M 2012 Liver-directed therapies in patients with advanced neuroendocrine tumors. Journal of the National Comprehensive Cancer Network 10 765–774. (10.6004/jnccn.2012.0076) 22679118

[bib31] Kahil ME Brown H & Fred HL 1964 The carcinoid crisis. Archives of Internal Medicine 114 26–28. (10.1001/ARCHINTE.1964.03860070072004) 14156067

[bib32] Kaltsas G Caplin M Davies P Ferone D Garcia-Carbonero R Grozinsky-Glasberg S Hörsch D Janson ET Kianmanesh R Kos-Kudla B, 2017 Enets consensus guidelines for the standards of care in neuroendocrine tumors: pre-and perioperative therapy in patients with neuroendocrine tumors. Neuroendocrinology 105 245–254. (10.1159/000461583) 28253514 PMC5637287

[bib33] Kaltsas GA Besser GM & Grossman AB 2004 The diagnosis and medical management of advanced neuroendocrine tumors. Endocrine Reviews 25 458–511. (10.1210/ER.2003-0014) 15180952

[bib34] Koumarianou A Kaltsas G Kulke MH Oberg K Strosberg JR Spada F Galdy S Barberis M Fumagalli C Berruti A, 2015 Temozolomide in advanced neuroendocrine neoplasms: pharmacological and clinical aspects. Neuroendocrinology 101 274–288. (10.1159/000430816) 25924937

[bib35] Kvols LK Moertel CG O’Connell MJ Schutt AJ Rubin J & Hahn RG 1986 Treatment of the malignant carcinoid syndrome. New England Journal of Medicine 315 663–666. (10.1056/NEJM198609113151102) 2427948

[bib36] Martin RCG Scoggins CR & McMasters KM 2010 Safety and efficacy of microwave ablation of hepatic tumors: a prospective review of a 5-year experience. Annals of Surgical Oncology 17 171–178. (10.1245/S10434-009-0686-Z) 19707829

[bib37] Murren JR & Buzaid AC 1989 The role of interferons in the treatment of malignant neoplasms. Yale Journal of Biology and Medicine 62 271–290. 2479178 PMC2589121

[bib38] Norton JA Warren RS Kelly MG Zuraek MB Jensen RT Fahey TJ Thompson NW Snyder SK Pasieka JL Mitchell B, 2003 Aggressive surgery for metastatic liver neuroendocrine tumors. Surgery 134 1057–1063. (10.1016/j.surg.2003.07.025) 14668741

[bib39] Öberg K 2001 Chemotherapy and biotherapy in the treatment of neuroendocrine tumours. Annals of Oncology 12(Supplement 2) S111–S114. (10.1093/annonc/12.suppl_2.s111) 11762335

[bib40] Palade E Günter J Gomez JMM Wellner UF Schmid S Wiesemann S & Passlick B 2019 Morbidity, mortality and long-term outcome of lung cancer resections performed in palliative intent. Journal of Thoracic Disease 11 4308–4318. (10.21037/jtd.2019.09.61) 31737316 PMC6837972

[bib41] Pavel ME Baudin E Öberg KE Hainsworth JD Voi M Rouyrre N Peeters M Gross DJ & Yao JC 2017 Efficacy of everolimus plus octreotide LAR in patients with advanced neuroendocrine tumor and carcinoid syndrome: final overall survival from the randomized, placebo-controlled phase 3 RADIANT-2 study. Annals of Oncology 28 1569–1575. (10.1093/annonc/mdx193) 28444114 PMC7360141

[bib42] Pavel ME Hainsworth JD Baudin E Peeters M Hörsch D Winkler RE Klimovsky J Lebwohl D Jehl V Wolin EM, 2011 Everolimus plus octreotide long-acting repeatable for the treatment of advanced neuroendocrine tumours associated with carcinoid syndrome (RADIANT-2): a randomised, placebo-controlled, phase 3 study. Lancet 378 2005–2012. (10.1016/S0140-6736(1161742-X) 22119496

[bib43] Pearman TP Beaumont JL Cella D Neary MP & Yao J 2016 Health-related quality of life in patients with neuroendocrine tumors: an investigation of treatment type, disease status, and symptom burden. Supportive Care in Cancer 24 3695–3703. (10.1007/S00520-016-3189-Z) 27029477

[bib44] Rapport MM Green AA & Page IH 1948 Crystalline serotonin. Science 108 329–330. (10.1126/SCIENCE.108.2804.329) 17748034

[bib45] Rinke A Müller HH Schade-Brittinger C Klose KJ Barth P Wied M Mayer C Aminossadati B Pape UF Bläker M, 2009 Placebo-controlled, double-blind, prospective, randomized study on the effect of octreotide LAR in the control of tumor growth in patients with metastatic neuroendocrine midgut tumors: a report from the PROMID Study Group. Journal of Clinical Oncology 27 4656–4663. (10.1200/JCO.2009.22.8510) 19704057

[bib46] Rosenau J Bahr MJ Von Wasielewski R Mengel M Schmidt HHJ Nashan B Lang H Klempnauer J Manns MP & Boeker KHW 2002 Ki67, E-cadherin, and p53 as prognostic indicators of long-term outcome after liver transplantation for metastatic neuroendocrine tumors. Transplantation 73 386–394. (10.1097/00007890-200202150-00012) 11884935

[bib47] Saavedra C Barriuso J Mcnamara MG Valle JW & Lamarca A 2019 Spotlight on telotristat ethyl for the treatment of carcinoid syndrome diarrhea: patient selection and reported outcomes. Cancer Management and Research 11 7537–7556. (10.2147/CMAR.S181439) 31496810 PMC6690650

[bib48] Strosberg JR Cheema A & Kvols LK 2011 A Review of systemic and liver-directed therapies for metastatic neuroendocrine tumors of the gastroenteropancreatic tract. Cancer Control 18 127–137. (10.1177/107327481101800207) 21451455

[bib49] Sutton R Doran HE Williams EMI Vora J Vinjamuri S Evans J Campbell F Raraty MGT Ghaneh P Hartley M, 2003 Surgery for midgut carcinoid. Endocrine-Related Cancer 10 469–481. (10.1677/erc.0.0100469) 14713260

[bib50] Svejda B Kidd M Giovinazzo F Eltawil K Gustafsson BI Pfragner R & Modlin IM 2010 The 5-HT(2B) receptor plays a key regulatory role in both neuroendocrine tumor cell proliferation and the modulation of the fibroblast component of the neoplastic microenvironment. Cancer 116 2902–2912. (10.1002/CNCR.25049) 20564397

[bib51] Tapia Rico G Li M Pavlakis N Cehic G & Price TJ 2018 Prevention and management of carcinoid crises in patients with high-risk neuroendocrine tumours undergoing peptide receptor radionuclide therapy (PRRT): literature review and case series from two Australian tertiary medical institutions. Cancer Treatment Reviews 66 1–6. (10.1016/j.ctrv.2018.03.002) 29602040

[bib52] Tellez MR Mamikunian G O’Dorisio TM Vinik AI & Woltering EA 2013 A single fasting plasma 5-HIAA value correlates with 24-hour urinary 5-HIAA values and other biomarkers in midgut neuroendocrine tumors (NETs). Pancreas 42 405–410. (10.1097/MPA.0b013e318271c0d5) 23160483

[bib53] Wang R Zheng-Pywell R Chen HA Bibb JA Chen H & Rose JB 2019 Management of gastrointestinal neuroendocrine tumors. Clinical Medicine Insights. Endocrinology and Diabetes 12 1179551419884058. (10.1177/1179551419884058) 31695546 PMC6820165

[bib54] Weickert MO Kaltsas G Hörsch D Lapuerta P Pavel M Valle JW Caplin ME Bergsland E Kunz PL Anthony LB, 2018 Changes in weight associated with telotristat ethyl in the treatment of carcinoid syndrome. Clinical Therapeutics 40 952–962.e2. (10.1016/j.clinthera.2018.04.006) 29724499

[bib55] Wolin EM 2012 The expanding role of somatostatin analogs in the management of neuroendocrine tumors. Gastrointestinal Cancer Research 5 161–168. 23112884 PMC3481148

[bib56] Wolin EM Jarzab B Eriksson B Walter T Toumpanakis C Morse MA Tomassetti P Weber MM Fogelman DR Ramage J, 2015 Phase III study of pasireotide long-acting release in patients with metastatic neuroendocrine tumors and carcinoid symptoms refractory to available somatostatin analogues. Drug Design, Development and Therapy 9 5075–5086. (10.2147/DDDT.S84177) PMC4562767 26366058

[bib57] Yao JC Hassan M Phan A Dagohoy C Leary C Mares JE Abdalla EK Fleming JB Vauthey JN Rashid A, 2008 One hundred years after “carcinoid”: epidemiology of and prognostic factors for neuroendocrine tumors in 35,825 cases in the United States. Journal of Clinical Oncology 26 3063–3072. (10.1200/JCO.2007.15.4377) 18565894

[bib58] Yao JC Shah MH Ito T Bohas CL Wolin EM Van Cutsem E Hobday TJ Okusaka T Capdevila J de Vries EGE, 2011 Everolimus for advanced pancreatic neuroendocrine tumors. New England Journal of Medicine 364 514–523. (10.1056/NEJMoa1009290) 21306238 PMC4208619

[bib59] Zandee WT Brabander T Blazević A Minczeles NS Feelders RA De Herder WW & Hofland J 2021 Peptide receptor radionuclide therapy with 177Lu-DOTATATE for symptomatic control of refractory carcinoid syndrome. Journal of Clinical Endocrinology and Metabolism 106 e3665–e3672. (10.1210/clinem/dgab289) 33942075 PMC8372632

